# Draft Genome Sequence of the Endophyte *Paenibacillus* sp. Strain GM2FR Isolated from *Festuca rubra*

**DOI:** 10.1128/genomeA.00017-18

**Published:** 2018-02-08

**Authors:** Franziska Wemheuer, Bernd Wemheuer, Jacqueline Hollensteiner, Rolf Daniel, Anja Poehlein

**Affiliations:** a Genomic and Applied Microbiology & Göttingen Genomics Laboratory, Institute of Microbiology and Genetics, University of Göttingen, Göttingen, Germany; b Department of Crop Sciences, Agricultural Entomology, University of Göttingen, Göttingen, Germany

## Abstract

Here, we report the 7.4-Mb draft genome sequence of *Paenibacillus* sp. strain GM2FR, an endophytic bacterium isolated from aerial plant tissues of *Festuca rubra* L. Genome analysis revealed 6,652 coding gene sequences and several gene clusters involved in plant growth promotion, such as that for the siderophore bacillibactin.

## GENOME ANNOUNCEMENT

Plant-associated bacteria belonging to the genus *Paenibacillus* are able to promote plant growth and health (1, 2). Several *Paenibacillus* spp. are important antagonists of various important plant pathogens and pests (3, 4). The genome sequence of the endophyte strain *Paenibacillus* sp. GM2FR will facilitate further studies on the potential of these bacteria for agricultural applications.

We isolated *Paenibacillus* sp. GM2FR from surface-sterilized aerial tissues of healthy *Festuca rubra* plants. Genomic DNA was extracted using the MasterPure complete DNA purification kit (Epicentre, Madison, WI, USA). The obtained DNA was used to generate Illumina shotgun paired-end sequencing libraries. Sequencing was performed employing a MiSeq system and the MiSeq reagent kit version 3 (600 cycles), as recommended by the manufacturer (Illumina, San Diego, CA, USA). Quality filtering using Trimmomatic version 0.32 (5) resulted in 5,564,116 paired-end reads. The *de novo* genome assembly was performed with the SPAdes genome assembler version 3.8.0 (6). The assembly resulted in 16 contigs (>500 bp) and an average coverage of 154-fold. The assembly was validated and the read coverage determined with QualiMap version 2.1 (7).

The draft genome of strain GM2FR consisted of 7,416,573 bp, with an overall GC content of 49.56%. Gene prediction and annotation were performed using Prokka (Rapid Prokaryotic Genome Annotation) version 1.11 (8). The draft genome harbored 8 rRNA genes, 45 tRNA genes, 2,573 protein-encoding genes with functional predictions, and 4,079 genes coding for hypothetical proteins. Multilocus sequence typing (MLST) based on four housekeeping genes (*gapA*, *groEL*, *gyrA*, and *pgi*) was performed according to Iiyama et al. (9). Strain GM2FR clustered with *Paenibacillus vortex* (10).

An antiSMASH 3.0.5 (11) analysis predicted 51 potential gene clusters involved in secondary metabolite production. These included 48 clusters with no or low (≤20%) similarity to known clusters, such as a bacteriocin, terpene, and type III polyketide synthase (T3pks) gene clusters. In addition, a nonribosomal polyketide synthetase (NRPS) cluster was identified with 53% of the genes sharing similarity to a bacillibactin biosynthesis gene cluster. Bacillibactin is a catecholate-type siderophore produced by *Paenibacillus larvae* (12) as well as several *Bacillus* species (13). Siderophores play an important role in competition between microorganisms (13) and can enhance plant growth and health (12–14). Moreover, a gene cluster with 75% of genes exhibiting similarity to an ectoine biosynthetic gene cluster was identified. Ectoines are produced by bacteria as compatible solutes to maintain the stability and correct folding of their proteins under osmotic stress (15). It has been shown that ectoines can enhance the nitrogen supply to tobacco leaves by increasing transpiration and by protecting RuBisCO proteins from the deleterious effects of salt (16). In addition, an increased water uptake rate and, subsequently, an increased photosynthesis rate under salt stress were observed in ectoine transgenic tomato plants (17). Thus, *Paenibacillus* sp. GM2FR might be involved in plant growth health in its host plant.

### Accession number(s).

The whole-genome shotgun project has been deposited at DDBJ/ENA/GenBank under the accession number MKZM00000000. The version described here is version MKZM01000000.
